# Jamaica

**Published:** 1896-12

**Authors:** 


*Jamaica.* The disease recently investigated by Professor W. Williams is reported by him to be in character that of Texas fever, assuming a chronic form, transmitted from animal to animal and from place to place by ticks. Great depression, languor, debility, and indifferent appetite, loss of rumination, emaciation, exceeding paleness of the visible mucous membranes, diarrhœa, pulse from 65 to 120 per minute, urine pale and containing but a small amount of albumin, breathing disturbed, shrunken appearance, and staggering gait. In the past two years two thousand cattle have died. For stamping out the tick plague stringent quarantine regulations, and, for a time, prohibition of all importing of animals to the island, were recommended by Williams.

